# The effect of the begining time of the rehabilitation program in stroke patients on functional ambulation and stroke related complications

**DOI:** 10.4314/ahs.v24i4.35

**Published:** 2024-12

**Authors:** Gülseren Demir Karakılıç, Esra Şahingöz Bakırcı, Ferda Büyük

**Affiliations:** Department of Physical Medicine and Rehabilitation, Yozgat City Hospital, Yozgat, Turkey

**Keywords:** Stroke, rehabilitation initiation time, rehabilitation period, functional recovery, stroke-related complications

## Abstract

**Objectives:**

The aim of this study was to reveal the effect of rehabilitation initiation time on the functional status and complications encountered during the rehabilitation process in stroke patients who underwent rehabilitation program in the physical medicine and rehabilitation clinic of our hospital.

**Material and methods:**

Patients who received outpatient and inpatient rehabilitation treatment with the diagnosis of acute stroke in the Physical Medicine and Rehabilitation Clinic of Yozgat City Hospital between January 2017 and August 2022 were screened. Patients with acute stroke and had a file record were included in the study.

Demographic information such as age, gender, comorbid diseases; type and direction of stroke, time from stroke until the rehabilitation program begins, total rehabilitation period, stroke-related complications, Brunnstrom stages, and functional ambulation scale scores of stroke patients were recorded

**Results:**

A total of 314 patients were included in this study and evaluated improvement in the functional ambulation scale scores according to the beginning time of the rehabilitation program. When the patients were grouped as 0-30 days, 31-60 days, and 61 days or more according to the time elapsed until the rehabilitation program, a significant difference was found in terms of the increase in FAS scores both in the pairwise comparisons and the comparison of all three groups together (p<0.001 for all comparisons). There was a moderate positive correlation between the total number of rehabilitation sessions and improvement in FAS scores (Rho=.316, p<0.01). Complications were observed in almost all patients in our study (97.7%). The comparison of the groups for stroke complications revealed that the frequency of depression, urinary tract infection, urinary incontinence, decubitus ulcer, dysphagia, convulsion, falling and aphasia was lower in the 0-30 days group compared to the other groups (p<0.01). There was also no significant correlation between the presence of stroke complications and improvement in FAS score (p>0.05).

**Conclusion:**

Contrary to popular belief, we showed that early rehabilitation does not increase complications and has a positive effect on function. Despite the perception that complications would negatively affect function, there was no significant relationship between the presence of stroke complications and the improvement in FAS score. We found that the rehabilitation program applied in the early period after stroke is effective and reliable.

## Introduction

Stroke, which ranks first among the diseases that cause disability in adults, is the second most common cause of death in the world after cardiovascular diseases and cancer, and is one of the important health problems due to its long-term disability^1^,^2^. The goal in stroke rehabilitation; it is to provide the highest level of functional independence and increase the quality of life of the individual despite the existing inadequacies 3. Although neurological recovery in stroke is mostly in the first 3 months, it is known that it can continue more slowly until the first 6 months, and very rarely up to 1 year^4^. 80% of motor recovery occurs within the first 3 to 6 months^5^. Rehabilitation of patients after stroke is of great importance in terms of reducing mortality, disability and the need for institutional care in the long term. Since complications related to immobilization occur more frequently and earlier after stroke, physical activities such as sitting in bed, standing and walking are recommended from the early period and it is thought that these will accelerate recovery^6^.

145 patients in a case-control study conducted by Paolucci S et al. were divided into 3 groups as short (first 20 days), medium (21-40 days), long (41-60 days) according to the time elapsed until the initiation of rehabilitation after stroke and the effect of rehabilitation onset time on prognosis was evaluated with the Barthel index. Significantly higher treatment efficacy was found in those who received treatment in the first 20 days compared to the others 7. 435 patients in another retrospectively study conducted by Salter K et al., were divided into 5 subgroups as 5-30, 31-60, 61-90, 91-120 and 121-150 days according to the duration of rehabilitation after stroke, and early and late treatment was given. The effects on functional independence and length of hospital stay were investigated. As a result of the study, it was observed that patients who applied to rehabilitation within the first 30 days had more functional gain and shorter hospital stay^8^. Serce A et al., divided 42 stroke patients into 3 groups according to the times of initiation of rehabilitation i.e during the first 9 days (Group 1), between days 10 and 14 (Group 2) and between days 15 and 21 (Group 3) and patients were assessed before the treatment, 4th weeks and 3th months. They stated that an early intensive and versatile rehabilitation program is effective for motor and functional recovery in ischemic stroke patients 9.

Complications after stroke have been reported at rates ranging from 40 to 96% in different studies. Complication development; affects mortality and rehabilitation process. Deaths occurring in the first days are usually due to cerebral edema and transtenterial herniation. Deaths in the subacute period are usually occur as a result of stroke complications. For this reason, it is very important to know the complications that may develop after stroke, and to recognize and treat them in the early period^10^,^11^. It has been reported that depression and speech disorders are the main causes of stroke-related complications in disability due to stroke. It has been stated that 3-4% of health expenditures in developed countries occur due to stroke^12^. Complications encountered during the treatment process of stroke patients further increase the health expenditures related to stroke. Post-stroke medical complications delays or disrupts recovery, prolongs hospital stay, and impairs functional results. The frequency of complications observed in studies has been reported to be between 44-95%^13^-^14^. Examining stroke-related complications and related factors is important for the treatment and prevention of complications, since they are common, closely related to the treatment process, and increase health costs.

Since complications related to immobilization occur more frequently and earlier after stroke, physical activities such as sitting in bed, standing and walking are recommended from the early period and it is thought that these will accelerate recovery. In conclusion; How often and how long the out-of-bed mobilization of patients will be after stroke, and when and at what dose rehabilitation should be started are still unanswered questions^17^. The aim of this study was to reveal the effect of rehabilitation initiation time on the functional status and complications encountered during the rehabilitation process in stroke patients who underwent rehabilitation program between 2017 and 2023 in the physical medicine and rehabilitation clinic of our hospital.

## Material and methods

### Study Design

This retrospective study was conducted between January 2017 and January 2023. The study protocol was approved by the Ankara City Hospital No. 2 Clinical Research Ethics Committee Presidency (E.Kurul-E2-22-2355).

### Patients

Patients who received outpatient and inpatient rehabilitation treatment with the diagnosis of acute stroke in the Physical Medicine and Rehabilitation Clinic of Yozgat City Hospital between January 2017 and August 2022 were screened in the study. Patients with acute stroke and had a file record were included in the study. Patients with chronic stroke and without a file record were not included in the study.

Demographic information such as age, gender, comorbid diseases; type and direction of stroke, time from stroke until the rehabilitation program begins, total rehabilitation period, stroke-related complications, Brunnstrom stages, and functional ambulation scale scores of stroke patients were recorded.

### Outcome measures

Brunnstrom staging: The motor functions of the patients were evaluated using the Brunnstrom motor staging method. Brunnstrom motor staging, which was evaluated in 3 different regions as upper and lower extremities and hand, was scored between 1- 6 for each region before treatment.

Functional ambulation scale (FAS): was used to determine the ambulation levels of the patients. Ambulation level was evaluated in 6 categories, including FAS 0-5 before and after treatment.

### Statistical analysis

Data were analyzed with SPSS 25.0 (IBM Co®. USA). Kolmogorov-Smirnov test was used for normality analysis. Categorical data were expressed as numbers and percentages (%). Numerical variables without parametric distribution were presented as the median and interquartile range (IQR 25-75 percentile). The Chi-square test with Yate's correction was used to compare categorical variables between groups. Mann-Whitney U test and Kruskal-Wallis test was used to compare numerical variables with non-parametric distribution between two or three groups respectively. Spearman correlation analysis was used to determine the factors associated with the improvement of FAS scores. P<0.05 was accepted as statistical significance.

## Results

Demographic data and clinical features of the patients are shown in Table 1.

**Table 1:** Demographic data and clinical features of the patients

		(N/%) (Total=314) Median (IQR:25^th^-75^th^)
Age		65.0(58.0-73.0)
Sex	Female	152 (48,4)
	Male	162 (51,5)
Side of stroke	Right	141 (44,9)
	Left	173 (55,1)
Type of stroke	Ischemic	286 (91,1)
	Hemorrhagic	28 (8,9)
Recurrent stroke	No	278 (88,5)
	Yes	36 (11,5)
Comorbidities*	Hypertension	214 (68,2)
	Diabetes mellitus	139 (44, 3)
	Cardiac disease	45 (14,3)
	Other systemic	9 (2,9)
	diseases	
Outpatient or inpatient	Outpatient	85 (27,1)
rehabilitation program	Inpatient	229 (72,9)
Time elapsed after stroke		30.0 (23.0-45.0)
Total number of rehabilitation sessions		60.0 (30.0-60.0)

* *Shows the total number of comorbidities*

***IQR:*** *Interquartile range*

Echocardiography examination was normal in 81.8% (257/314) of the patients, and pathologic findings were detected in 18.2% (57/314). Carotid doppler USG was normal in 68.2% (214/314) of the patients, 29% (91/314) had atheroma plaque and 2.9% (9/314) had carotid artery stenosis. The frequency of stroke-related complications, pre-treatment Brunnstrom values, and pre-and post-treatment FAS scores are given in Table 2.

**Table 2:** Stroke-related complications, Brunnstrom stages, and functional ambulation scale scores of stroke patients

		(N/%) (Total=314) Median (IQR:25^th^-75^th^)
Shoulder pain		297 (94,6)
Depression		182 (58,0)
Aphasia		61 (19,4)
Incontinence		278 (88,5)
Dysphagia		117 (37,3)
LRTI		154 (49,0)
UTI		251 (79,9)
DVT		10 (3,2)
Peripheral arterial embolism		6 (1,9)
Decubitus ulcer		21 (6,7)
		30 (9,6)
Convulsion		39 (12,4)
Falling		3.0 (2.0-3.0)
Brunnstrom staging (Pre-treatment)	Upper extremity	3.0 (2.0-3.0)
	Hand	3 0 (3 0-3 0)
	Lower extremity	0.0 (0.0-1.0)
FAS score	Pre-treatment	2.0 (1.0-3.0)
	Post-treatment	

***IQR:** Interquartile range, **LRTI:** Lower respiratory tract infection, **UTI:** Urinary tract infection, **DVT:** Deep vein thrombosis, **FAS:** Functional ambulation scale*

There were no significant differ ences between ischemic and hemorrhagic stroke groups in terms of age (p=0.135), gender (p=0.330), outpatient or inpatient treatment condition (p=0.656), and the side of stroke (p=0.845). Rehabilitation onset time was longer in the hemorrhagic stroke group (P=0.022). While there was no significant difference in Brunnstrom stages of the upper extremity, hand, lower extremity, and FAS scores before treatment (p>0.05), FAS scores after treatment were lower in the hemorrhagic stroke group (p=0.029). There was no significant difference between the groups regarding improvement in FAS scores (p=0.074).

When the patients were grouped as 0-30 days, 31-60 days, and 61 days or more according to the time elapsed until the rehabilitation program, a significant difference was found in terms of the increase in FAS scores both in the pairwise comparisons and the comparison of all three groups together (p<0.001 for all comparisons) (Table 3). The comparison of the groups for stroke complications revealed that the frequency of depression (p=0.113), urinary tract infection (p=0.102), decubitus ulcer (p=0.058), convulsion (p=0.072), falls (p=0.296) and aphasia was lower in the 0-30 days group compared to the other groups (p<0.01). Urinary incontinence frequency was lower in the 0-30 days group than in the 31-60 days group (p=0.018). The frequency of dysphagia was also lower in the 0–30-day group compared to the other two groups (p<0.01). Lower respiratory tract infection frequency was lower in the 0-30 group than in the 31-60 group (p<0.001). Since the expected counts for the Chi-square test were not obtained for shoulder pain, deep vein thrombosis, and peripheral arterial embolism, no comparison could be made between the groups for these variables.

**Table 3:** Improvement in the functional ambulation scale scores according to the beginning time of the rehabilitation program

		Improvement in the FAS scores (Median (IQR:25^th^ - 75^th^)	p
Time to	0-30 days	2.0 (1.0-3.0)	<0.001*
start the rehabilitation	31-60 days	1.0 (1.0-2.0)	
program	61 days or more	1.0 (0.0-1.0)	

* p<0.001 was obtained for all pairwise comparisons and the comparison of three groups together.

***FAS:*** *Functional ambulation scale*

No significant correlation was found between age, gender, stroke type, presence of recurrence and receiving outpatient or inpatient treatment, and improvement in the FAS scores. There was a moderate positive correlation between the total number of rehabilitation sessions and improvement in FAS scores (Rho=.316, p<0.01) (Table 4). There was also no significant correlation between the presence of stroke complications and improvement in FAS score (p>0.05)

**Table 4:** Correlation analysis of factors associated with an improvement in functional ambulation scale scores

		Improvement of FAS scores
Age	Rho	-.043
	P	0.444
Sex	Rho	-.026
	P	0.647
Type of stroke (ischemic/hemorrhagic)	Rho	-.101
	P	0.074
Presence of recurrence stroke	Rho	.040
	P	0.479
Outpatient or inpatient treatment	Rho	-.024
	P	0.672
Total number of rehabilitation sessions	Rho	**.316**
	P	**<0.001**

***FAS:** Functional ambulation scale*

## Discussion

A total of 314 patients were included in this study and evaluated improvement in the functional ambulation scale scores according to the beginning time of the rehabilitation program. When the patients were grouped as 0-30 days, 31-60 days, and 61 days or more according to the time elapsed until the rehabilitation program, a significant difference was found in terms of the increase in FAS scores both in the pairwise comparisons and the comparison of all three groups together (p<0.001 for all comparisons). There was a moderate positive correlation between the total number of rehabilitation sessions and improvement in FAS scores (Rho=.316, p<0.01).

The frequency and duration of out-of-bed mobilization of patients after stroke, and when and at what dose rehabilitation should be initiated are still unanswered questions^17^. Australian guidelines recommend initiating rehabilitation on the first day after stroke, the United States as early as possible after medical stability has been established, and European guidelines simply start as early as possible without specifying time 18. Early rehabilitation cannot be performed very often in patients with hemorrhagic stroke due to concerns about blood pressure changes during early exercise and mobilization and opinions that increase the bleeding volume in patients with intracerebral hemorrhage and enlarge the penumbra area due to the decrease in blood flow in the middle cerebral artery with mobilization in patients with ischemic lesions^19^. Consistent with the literature rehabilitation onset time was longer in the hemorrhagic stroke group (P=0.022).

The most comprehensive study on early rehabilitation after stroke in the literature is the AVERT (Avery Early Rehabilitation Trial) study. This study argues that early mobilization from the first 24 hours after stroke is safe and beneficial 19. It was determined that 32 patients who were mobilized and rehabilitated within the first 24 hours after stroke were more independent at the 3rd month compared to the control group who received standard care in a randomized controlled study conducted by Langhorne et al.^20^ 435 patients were divided into 5 subgroups as 5-30, 31-60, 61-90, 91-120 and 121-150 days, according to the starting time of rehabilitation after stroke in another retrospective study conducted by Salter K et al. and It has been reported that patients who applied to rehabilitation within the first 30 days had higher functional gains and shorter hospital stays^8^. Consistent with the literature, the shorter the time until the rehabilitation program started, the higher the increase was in FAS scores p<0.001). Unlike the literature, the total duration of the rehabilitation program applied in patients with a short rehabilitation start time was not short (p>0.001). On the contrary there was a moderate positive correlation between the total number of rehabilitation sessions and improvement in FAS scores (Rho=.316, p<0.01)

Complications were observed in 85% of 311 stroke patients in a multicenter study by Langhorne et al. Complications observed in this study were confusion (56%), pain (34%), falling (25%), urinary tract infection (24%) and lung infections (22%), and depression (16%) [21]. 81 stroke patients were evaluated during the rehabilitation process in the study conducted by Civelek et al. and at least one complication was observed in 88.9% of the patients. Among these complications, the most common ones are urinary tract infection (48.1%), shoulder pain (37%), insomnia (37%), depression (32.1%), musculoscletal pain other than shoulder (32.1%), arrhythmia (16%) and pneumonia (13.6%) 10. 232 stroke patients were evaluated in a multicenter study conducted by Kuptniratsaikul et al. and at least one complication was observed in 71% of the patients including spasticity (41.6%), shoulder subluxation (37.3%) and musculoscletal pain^22^. Similar to these studies complications were observed in almost all patients in our study (97.7%). The most common complications are shoulder pain (94.6%), incontinence (88.5%), urinary tract infection (79.9%), depression (58%), lower respiratory tract infection (49%), dysphagia (37.3%), aphasia (19.4%) and falling (12.4%).

Early post-stroke rehabilitation has been shown to help reduce complications and functional barriers remaining after stroke. The reduction in functional disability as well as the incidence of complications results in a higher quality of life for stroke survivors and a reduction in potentially expensive long-term care cost^23^,^24^. Savas et al. divided the patients into 2 groups as early admission (<30 days) and late admission (>30 days) groups according to the time elapsed between stroke event and admission to rehabilitation, and they found that the number of complications in the late admission group was higher than the early admission group 25. Similar to previous studies, in our study the comparison of the groups for stroke complications revealed that the frequency of depression, urinary tract infection, urinary incontinence, decubitus ulcer, dysphagia, convulsion, falling and aphasia was lower in the 0-30 days group compared to the other groups (p<0.01). There was also no significant correlation between the presence of stroke complications and improvement in FAS score (p>0.05).

## Limitations

The limitations of our study were the retrospective nature of our study, the absence of Brunnstrom staging after treatment, and the inability to evaluate upper extremity functions.

## Conclusions

Many studies suggest starting rehabilitation in the early period after stroke, but studies on the effect of the rehabilitation program started in the early period on function and complications are insufficient. Contrary to popular belief, we showed that early rehabilitation does not increase complications and has a positive effect on function. Despite the perception that complications would negatively affect function, there was no significant relationship between the presence of stroke complications and the improvement in FAS score. We found that the rehabilitation program applied in the early period after stroke is effective and reliable.
